# Locus coeruleus vulnerability to tau hyperphosphorylation in a rat model

**DOI:** 10.1111/acel.14405

**Published:** 2024-11-09

**Authors:** Tamunotonye Omoluabi, Zia Hasan, Jessie E. Piche, Abeni R. S. Flynn, Jules J. E. Doré, Susan G. Walling, Andrew C. W. Weeks, Touati Benoukraf, Qi Yuan

**Affiliations:** ^1^ Biomedical Sciences, Faculty of Medicine Memorial University of Newfoundland St. John's Newfoundland Canada; ^2^ Department of Psychology, Faculty of Arts & Science Nipissing University North Bay Ontario Canada; ^3^ Department of Psychology, Faculty of Science Memorial University of Newfoundland St. John's Newfoundland Canada

**Keywords:** electron microscopy, locus coeruleus, L‐type calcium channel, mitochondria, single nuclei RNA sequencing, tau

## Abstract

Post‐mortem investigations indicate that the locus coeruleus (LC) is the initial site of hyperphosphorylated pretangle tau, a precursor to neurofibrillary tangles (NFTs) found in Alzheimer's disease (AD). The presence of pretangle tau and NFTs correlates with AD progression and symptomatology. LC neuron integrity and quantity are linked to cognitive performance, with degeneration strongly associated with AD. Despite their importance, the mechanisms of pretangle tau‐induced LC degeneration are unclear. This study examined the transcriptomic and mitochondrial profiles of LC noradrenergic neurons after transduction with pseudophosphorylated human tau. Tau hyperphosphorylation increased the somatic expression of the L‐type calcium channel (LTCC), impaired mitochondrial health, and led to deficits in spatial and olfactory learning. Sex‐dependent alterations in gene expression were observed in rats transduced with pretangle tau. Chronic LTCC blockade prevented behavioral deficits and altered mitochondrial mRNA expression, suggesting a potential link between LTCC hyperactivity and mitochondrial dysfunction. Our research provides insights into the consequences of tau pathology in the originating structure of AD.

## Graphical abstract

Hyperphosphorylated tau (ptau) leads to calcium overload via LTCC Cav1.2 channels (1), mitochondrial complex I deficit (2), increased mitochondrial fusion (3), which can be associated with reactive oxygen species (ROS) production. Ptau also impedes anterograde transport of distinct cargoes including mitochondria. (4) Together, these pathological changes lead to synaptic and axonal degeneration in the locus coeruleus neurons.

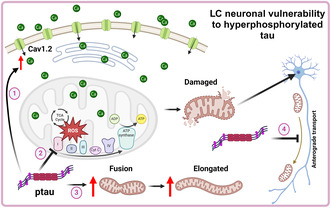

**Abbreviations**
AAV: adeno‐associated virus
Aβ: beta‐amyloid
AD: Alzheimer’s disease
DBH: dopamine beta‐hydroxylase
DIO: double‐inverted open reading frame
EPM: elevated plus maze
htau: human tau
htauE14: human tau pseudophosphorylated at 14 sites
IHC: immunohistochemistry
LC: locus coeruleus
LTCC: L‐type calcium channel
MBT: marble burying test
NFT: neurofibrillary tangle
PBS: phosphate buffered saline
qPCR: quantitative PCR
ROS: reactive oxygen species
SLR: spontaneous location recognition
SPT: sucrose preference test
TEM: transmission electron microscopy
TH: tyrosine hydroxylase

## 1 INTRODUCTION

Alzheimer's disease (AD) is a prevalent and debilitating condition. Despite its massive disease burden, much remains unknown regarding its biological course. Although much attention has been paid to the deposition of amyloid‐β (Aβ) plaques and neurofibrillary tangles (NFTs) as drivers of AD progression and symptomatology (Hardy & Higgins, 1992; Long & Holtzman, 2019; Scheltens et al., 2016), recent evidence suggests that the disease could begin much earlier than previously conceived. Braak and colleagues, after analyzing more than 2000 post‐mortem human brains, have characterized the spread of the soluble, persistently phosphorylated protein precursor of NFTs (henceforth referred to as pretangle tau) across the lifespan (Braak et al., 2011). Pretangle tau appears in the locus coeruleus (LC) as early as the first decade of life, from where it spreads to other areas involved in learning and memory (Braak et al., 2011), potentially leading to neurodegeneration in these structures later in life (Yoshiyama et al., 2007). Furthermore, LC neuronal degeneration itself plays a crucial role in cognitive decline and the progression of AD (Bueichekú et al., 2024; Kelly et al., 2017; Theofilas et al., 2017; Wilson et al., 2013). As such, pretangle tau toxicity in the LC may represent the earliest pathology leading to AD. Indeed, recent evidence suggests that pretangle tau, including its oligomer form, may exhibit greater toxicity than fully formed NFTs (Brunden et al., 2008; Congdon & Duff, 2008; Spires‐Jones & Hyman, 2014), making it an attractive target for therapeutic interventions aimed at preventing AD progression.

However, much remains unknown regarding the mechanisms by which pretangle tau leads to neurodegeneration and deficits in learning and memory in AD. One promising avenue of research involves the interaction between tau pathology and mitochondrial dysfunction, which becomes increasingly pronounced with age (Osellame et al., 2012). Tau overexpression and hyperphosphorylation disrupt microtubule assembly and anterograde transport (Götz & Ittner, 2009), potentially hindering the transport of mitochondria to synapses. Additionally, in a neuronal cell line, expression of a phosphorylation‐prone P301L mutant tau inhibits complex I of the electron transport chain, resulting in elevated reactive oxygen species (ROS) production and impaired mitochondrial morphology (Schulz et al., 2012). Furthermore, dysregulation of calcium homeostasis, mediated by abnormal tau phosphorylation and dysfunction of L‐type calcium channels (LTCCs), could exacerbate mitochondrial damage (Sanchez‐Padilla et al., 2014; Tracy et al., 2022).

Here we employed a pretangle tau rat model, closely mimicking the progression of preclinical tau pathology as outlined by Braak, by seeding a human pseudophosphorylated tau at 14 sites (htauE14), a pretangle tau mimic, in the rat LC (Ghosh et al., 2019; Omoluabi et al., 2021). Through a combination of behavioral assessments, single nuclei transcriptomic analyses, and investigations of mitochondrial morphology and mRNAs in LC neurons, we uncovered a link between pretangle tau and mitochondrial dysfunction. These findings underscore the potential significance of targeting calcium dysregulation of mitochondrial pathways as a therapeutic strategy for AD. Further research is warranted to unravel the full extent of these complex interactions and their therapeutic implications.

## 2 MATERIALS AND METHODS

### 2.1 Subjects & ethics statement

Mixed sex offspring of homozygous tyrosine hydroxylase (TH)‐CRE male breeders (Sage Labs) and Sprague–Dawley female rats (Charles River) were used in the study. Rats were housed in a standard 12‐h light–dark cycle environment (with light from 7 am–7 pm) and had ad libitum access to food and water, except for the day prior to the sucrose preference test (SPT). Rats assigned to the nimodipine chronic injection group were subjected to a modified light cycle (light from 2 am–2 pm) following adeno‐associated virus (AAV) infusion surgeries, which persisted until the conclusion of the experiments. All experimental procedures were ethically approved by the Institutional Animal Care Committee at Memorial University of Newfoundland and adhered to the guidelines outlined by the Canadian Council for Animal Care.

All procedures were designed to minimize pain and distress. We monitored rats closely following surgeries and use pain medication Meloxicam (0.5 mg/mL at 1 mg/kg) for surgery rats. Sufficient handling and habituation were conducted during behavioral experiments to minimize stress. Pentobarbital (80 mg/kg) was used to euthanize the rats for brain tissue.

### 2.2 Experimental design

**FIGURE 1:**
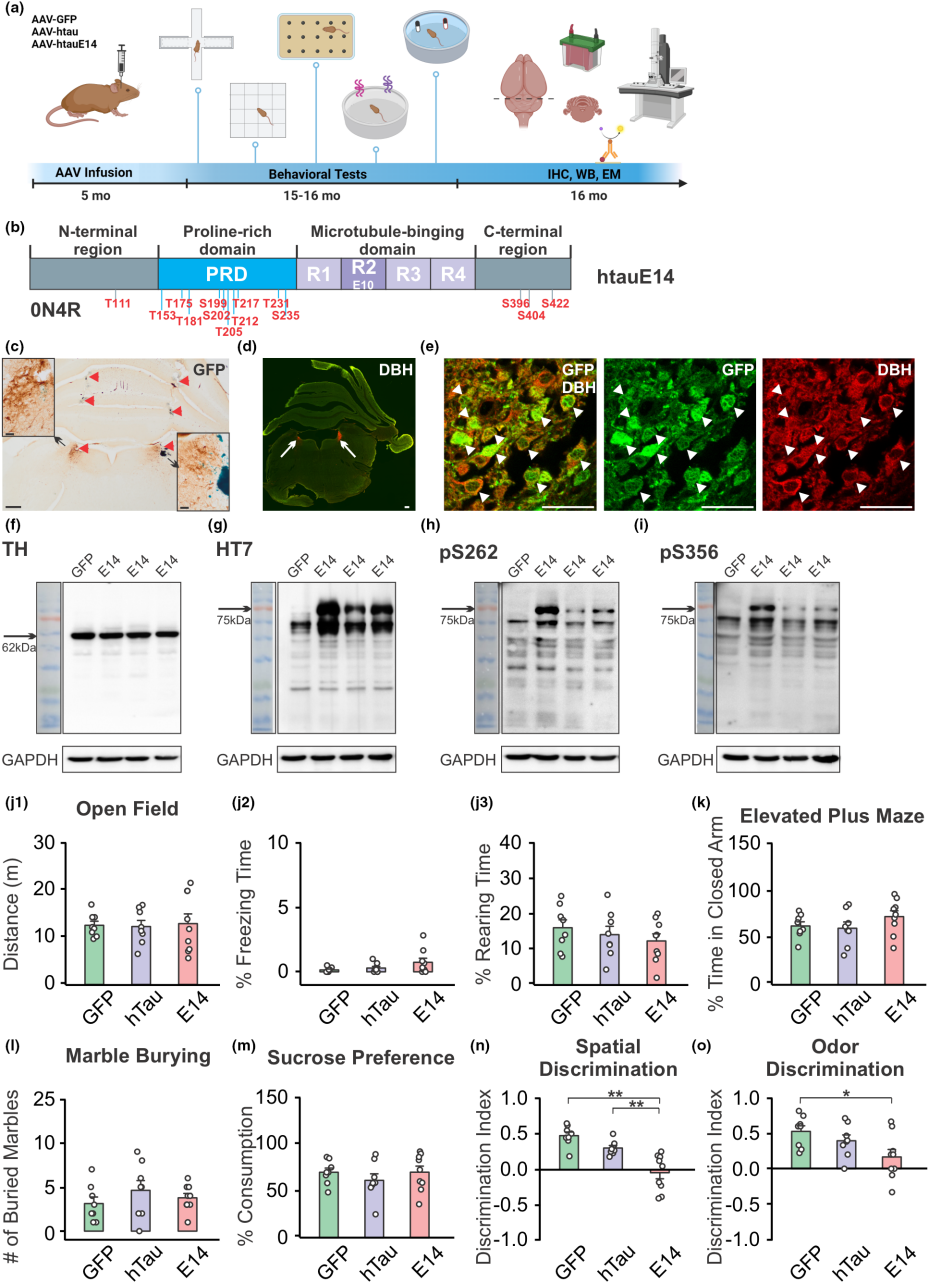
Human pretangle tau mimic in the rat locus coeruleus results in impaired spatial and olfactory discrimination learning. (a) Schematic diagram demonstrating the experimental flow. (b) Construct of htauE14. Human tau protein contains four domains as shown. The presence or absence of exons 2, 3 at the N‐terminal and exon 10 (E10) at the microtubule‐binding domain, form six isoforms of tau. We used 0N4R isoform of human tau with 14 sites of pseudophosphorylation, as shown. The majority of the phosphorylation sites are within the proline‐rich domain. (c) Example of AAV uptake in the LC, indexed by GFP staining. The inserts show enlargement of the LC. Red arrowheads indicate cannula tracks. (d). Example of a brainstem section containing dopamine β‐hydroxylase (DBH^+^) cells (red) in the LC. Scale bar, 500 μm. (e) GFP (green) and DBH (red) double‐labeled noradrenergic neurons in the LC. Scale bar, 50 μm. (f) Tyrosine hydroxylase (TH) expression in one GFP and three htauE14 (briefly E14) LCs. (g) Human tau HT7 expression in the htauE14 LC. (h, i) Expression of phosphorylated tau at S262 (h) and S356 (i) in the htauE14 LC. In panels (f–i) the molecular weight label is overlaid on the image, separated from the gel bands by a black box. (J1‐J3) Distance traveled (J1), percentage of time spent freezing (J2) and percentage of time spent rearing (J3) in an open‐field test. (k) Percentage of time spent in the close arm of an elevated maze. (l) Number of marbles buried. (m) Percentage of sucrose water consumed. (n) Discrimination index in a spontaneous location recognition test. (o) Discrimination index in an odor discrimination test. *N* = 3F/5M (GFP), 7F/1M (htau) and 4F/5M (htauE14). *p < 0.05; **p < 0.01.

Figure 1a illustrates the experimental flow. Rats were randomly assigned and received infusions of AAV carrying human tau (htau) pseudophosphorylated at 14 sites (htauE14) (AAV9‐rEF1a‐DIO‐EGFP‐htauE14), htau (AAV9‐rEF1a‐DIO‐EGFP‐htau), or control GFP AAV (AAV9‐rEF1a‐DIO‐EGFP) into the LC at 5 months of age. The EGFP‐htauE14 expression cassette was inserted under a double‐inverted open reading frame (DIO) for CRE‐dependent expression in noradrenergic LC cells in TH‐CRE rats. At 15 months of age, rats underwent a battery of behavioral tasks to evaluate general behavior and cognitive function. Subsequently, at 16 months, a subset of animals was perfused for electron microscopy examination of mitochondrial morphology in the LC. Another cohort was sacrificed to assess viral transduction, tau phosphorylation, and LTCC Cav1.2 expression in the LC. Finally, another cohort of rats were injected with AAVs at 3 months of age. These rats were sacrificed without behavioral assessment at 11 months of age, and LC tissue was extracted and subjected to single nuclei RNA sequencing (Figure 3a).

For the assessment of chronic LTCC blockade with nimodipine, two age cohorts were employed (Figure 4a). In one cohort, rats were infused with AAVs carrying htauE14 or control GFP at 3 months of age. Subsequently, rats were randomly assigned to nimodipine or vehicle injections at 7 months of age for a duration of 6 weeks. Behavioral testing was conducted following nimodipine or vehicle administration, after which LC tissue was harvested and subjected to qPCR to measure expression levels of LTCC subunits and mitochondrial mRNA. In the other cohort, rats were infused with AAVs at 14–15 months of age and subsequently received nimodipine or vehicle injections 2 months later. This was followed by behavioral testing at 18–19 months of age. Animals were sacrificed at 20 months of age and LC mRNA was isolated for gene expression assessment using qPCR.

### 2.3 AAV infusion surgeries

Under isoflurane anesthesia, rats were positioned in a stereotaxic apparatus and infused with either AAV9‐rEF1a‐DIO‐EGFP‐htauE14 (2.26E+ 13 vg/ml, Virovek), AAV9‐rEF1a‐DIO‐EGFP‐htau (2.35E+ 13vg/ml), or control AAV9‐rEF1a‐DIO‐EGFP (1.5E+ 13 vg/ml) into the LC. htauE14 was generated by mutating 14 S/P or T/P amino acid residues of the 0N4R human tau isoform, to glutamate (Hoover et al., 2010) (Figure 1b). Each LC received 1 μL of AAV solution combined with 0.4 μL blue fluorescent beads (0.1%; ThermoFisher Scientific) via an infusion pump and a guide cannula angled at 20° parasagittally, caudal to the coronal plane. The coordinates for LC infusion were 11.8–12.2 mm posterior, 1.3 mm bilateral, and 6.3 mm ventral relative to the bregma sequence (Ghosh et al., 2019).

### 2.4 Behavioral experiments

All behvioural tasks were conducted blindly and animals were randomly assigned to groups. All tasks were completed in the same sequence in order to maintain control for all cohorts. The animals were always tested in the same order within the cohorts, as well as at similar times for the mutiple day tasks.

### 2.5 Open field test

Rats underwent a single 10‐min trial to explore an open field measuring 60 × 60 × 40.5 cm^3^, with their activity recorded using ANY‐Maze software (Stoelting). Parameters such as distance traveled, time spent rearing (including both free and supported rearing), and time spent freezing were recorded during the trial and subsequently analyzed offline by an experimenter blind to the experimental conditions. Freezing behavior was characterized by the absence of body movement except for breathing, while rearing was defined as lifting both front paws off the ground.

### 2.6 Elevated plus maze

To evaluate anxiety‐like behavior, the animals were placed in an elevated plus‐shaped maze (EPM) elevated 50 cm above the ground. The arms of the maze measured 50 × 10 cm^2^, with the two closed arms bordered by walls reaching a height of 38 cm along the sides and at the end of the arm. Throughout the 5‐min trial, the duration of time the animals spent in both the closed and open arms was recorded.

### 2.7 Marble burying test (MBT)

In the MBT, 16 identical dark‐blue marbles were arranged in a 4 × 4 grid on sanitized extra bedding within a clean cage. The animals were then observed in the cage via video recording for a duration of 10 min. The number of marbles buried at least 75 percent beneath the bedding was recorded. An increase in the number of marbles buried is indicative of heightened stress‐like symptoms (Archer et al., 1987). The impact of the htau and htauE14 on stress‐like symptoms across groups was assessed based on the number of marbles buried.

### 2.8 SPT

The SPT was conducted after withholding water from the animals for a full day. Each animal was provided with two identical water bottles: one containing ordinary tap water and the other containing a tap water‐based 0.1% sucrose solution. After 24 h, the bottles were removed from the cages and liquid consumed was measured. Throughout the test, the animals had unrestricted access to the bottles. The ratio of sucrose consumed to total fluid consumed was calculated. A decrease in preference for sucrose indicates anhedonia, or the inability to experience pleasure (Liu et al., 2018).

### 2.9 Spontaneous location recognition (SLR)

In the SLR task, designed to assess long‐term memory, rats were initially allowed 10 min of exploration in an open arena measuring 60 × 60 × 40.5 cm^3^. Within this arena, three identical objects (labeled 1, 2, and 3) were positioned at specific locations. Objects 2 and 3 were situated 20 cm apart. During the subsequent testing phase, conducted 24 h later, rats were reintroduced into the same arena. This time, only two objects were present: one positioned at the same location as Object 1 (representing a familiar location), and the other placed midway between the previous positions of Objects 2 and 3 (representing a novel location). The discrimination ratio, calculated as the difference between the time spent investigating the objects at the novel and familiar locations, divided by the total time spent at both locations investigating the objects, was used to indicate animal's spatial learning performance.

### 2.10 Odor discrimination

To assess the discrimination of similar odors, rats underwent an odor detection and discrimination task. Perforated micro‐centrifuge tubes containing filter paper soaked in either 60 μL of odorant or mineral oil were used. The testing protocol involved a series of trials. The first three trials employed odorless mineral oil, followed by three trials using Odor 1 (O1, 1‐heptanol at a concentration of 0.001%). The final trial involved an odor mixture (O2) designed to have a similar scent to O1, comprising a blend of 1‐heptanol and 1‐octanol in a 1:1 ratio, also at a concentration of 0.001%. Each trial lasted for 50 s, with a 1‐min interval between trials. The trial commenced upon the first sniff by the subject. The testing process was recorded via video, and offline analysis involved measuring the duration of sniffing within a 1 cm radius around the odor tube. The discrimination index was calculated as the ratio of the difference in sniffing time between O2 and the third presentation of O1, divided by the total sniffing time: (tO2 – tO1–3)/(tO2 + tO1–3).

### 2.11 Nimodipine chronic injection

A separate cohort of animals received chronic subcutaneous nimodipine (5 mg/kg; dissolved in 50% DMSO and 50% PBS) or vehicle 5 days a week for 6 weeks leading up to the behavioral tests. Throughout the subsequent 2 weeks of behavioral testing, injections were administered concurrently. To prevent acute drug effects, injections were administered during the dark phase of the cycle, while behavioral testing occurred during the light phase, with an interval of 18 h between them.

### 2.12 Immunohistochemistry (IHC) and analysis

After decapitation, LC tissue was extracted and preserved in 20% sucrose in 0.1 M phosphate‐buffered saline (PBS). Coronal sections measuring 25 μm were obtained using a cryostat (HM550, Thermoscientific) and mounted on chrome‐gelatin coated slides. IHC procedures followed established protocols. Primary antibodies included dopamine β‐hydroxylase (DBH) (MAB308, Millipore‐Sigma, 1:2000), GFP (A11122, Invitrogen, 1:2000) and FP1 rabbit anti‐Cav1.2 antibody (1:2000) (Buonarati et al., 2017) in PBS with 0.2% Triton‐X and 2% goat serum. Following primary antibody incubation at 4°C for 1–3 nights, tissue sections were washed and then incubated with Alexa Fluor secondary antibodies (Invitrogen, 1:1000) at room temperature for 2 h. Subsequently, cover‐slips were applied using Fluoroshield Mounting Medium with 4′,6‐diamidino‐2‐phenylindole (ThermoFisher).

Fluorescence microscopy was performed using an EVOS M5000 imaging system (Thermo Fisher Scientific). Image analysis was conducted with ImageJ software. Light intensity and exposure parameters were standardized across all captured images. For Cav1.2 expression level, regions of interest were chosen randomly from 10 GFP^+^ somata, and integrated fluorescence intensity was measured and averaged for each slice. Within the LC, the numbers of positively stained DBH and GFP cells were quantified. Three sections within the same rostral to caudal range were analyzed from all animals and counts from the two hemispheres were averaged. Analysis was performed by experimenters who were blinded to the experimental conditions. Example images were acquired with a confocal microscope (Zeiss LSM 900).

### 2.13 Western blotting

Rats were removed from their cages and anesthetized with isoflurane prior to decapitation. Following decapitation, hindbrains from rats were rapidly frozen using flash‐freezing techniques and stored at −80°C. Cryosections of the LC, measuring 1500 μm per hemisphere, were obtained from the hindbrains using a 1 mm diameter puncture and immediately transferred into RNAse‐free 1.5 mL tubes placed on dry ice to maintain tissue integrity. Brain tissue processing followed established protocols (Crossley et al., 2024). Briefly, frozen brain samples were homogenized using the Precellys tissue homogenizer in lysis buffer (Thermo Scientific, Pierce RIPA Buffer, 89,901). EDTA‐free 1x phosphatase and protease inhibitors (Thermo Scientific, 87,786 and 78,420) were added during homogenization to prevent protein degradation. The homogenate was then centrifuged at 13,500 rpm for 30 min at 4°C. The supernatant was carefully aliquoted into new tubes for protein estimation.

Total protein concentration was determined using the standard Pierce BCA protein assay kit (Thermo Scientific, 23,225). Equal amounts of protein (20 μg) were loaded onto 10% SDS‐PAGE gels and subsequently transferred to Immobilon‐P Transfer PVDF membranes (Merck Millipore, IPVH00010). Following transfer, the membranes underwent a brief rinse with 1x low salt TBS‐T (composed of 150 mM NaCl, 1 M Tris Base, and 0.1% Tween 20) and were then blocked for 1 h at room temperature using 5% nonfat skim milk in TBS‐T. Subsequently, the membranes were incubated with the following antibodies for 2 h at room temperature: pTau S262 (AB92627, Abcam, 1:2000) or pTau S356 (AB75603, Abcam, 1:5000). Afterward, the membranes were rinsed twice for 5 min each in high salt TBS‐T (containing 500 mM NaCl, 1 M Tris Base, and 0.1% Tween 20), followed by one 5‐min rinse in low salt TBS‐T. They were then incubated for 1.5 h at room temperature with either horseradish peroxidase–labeled anti‐rabbit immunoglobulin G (IgG; 1:4000) or anti‐mouse IgG (1:4000; Thermo Fisher Scientific). Subsequently, the protein bands were visualized using chemiluminescent substrate (ThermoFisher Supersignal West PICO) and captured with a digital image scanner (ImageQuant LAS 4000). Quantification of the bands was performed using ImageJ software.

## 3 ELECTRON MICROSCOPY

### 3.1 Tissue processing

Transcardial perfusions were performed in a three‐step process as described previously by Connor et al., (2009). Phosphorylate‐buffered saline containing Heparin sodium (10 units/mL) was injected initially at a rate of 50 mL per min for 1 min. This was followed by a fixative solution consisting of 1% paraformaldehyde, 1.25% glutaraldehyde, and 0.02 mM CaCl2 in 0.12 M phosphate buffer (pH 7.3), perfused at 50 mL per min for 10 min, then at 25 mL per min for 20 min. Finally, a fixative solution with double the aldehyde concentration was perfused at 25 mL/min for 12 min. All solutions were warmed to thirty‐seven degrees Celsius and delivered using a peristaltic pump. Following perfusion, the animals were sealed in plastic bags and refrigerated at 4°C for 2 h.

Brains were serial sectioned at 700 μm using a Pelco 102 Vibratome according to bregma coordinates (Paxinos and Watson, 6th ed.) (Paxinos & Watson, 2009). The optimal 3–4 sections (Bregma −9.48 to −10.44) from the vibratome containing LC tissue were analyzed under the Olympus SZ61 dissecting microscope. The LC was dissected from each 700 μm section and placed into 0.1 M Sorensen's phosphate buffer solution. Tissue was post‐fixed with osmium, dehydrated, and then embedded in LR white resin and heat‐cured (Isotemp 500) for 48 h. Resin blocks were trimmed and semi‐thin sections (1000 nm) were generated using a glass knife and stained with Toluidine Blue O. Once subsequent trimming delineated the boundaries of the LC, ultra thin serial sections were cut at 60–65 nm from three distinct depths through the LC blocks using a diamond knife.

### 3.2 Imaging and analysis

Ultra‐thin sections were mounted on copper grids (300 × 300) and imaged using a Philips transmission electron microscope. Three cells were randomly selected from each depth, ensuring a clear nucleus and cell body not obscured by grid lines. Three neurons were randomly selected as per Nahirney and Tremblay (2021) (Nahirney & Tremblay, 2021). The neurons contained a large euchromatic nucleus with centrally located nucleoli, and a large primary dendrite leaving the cell body. Neurons are distinct from satellite cells (microglia and oligodendrocyte precursor cells) that have a smaller ovoid nucleus, and glial cells depict darker, granule‐dense cell bodies. Entire cell bodies were imaged at 2300X–10,500X depending on cell size. Subsequently, images of mitochondrial profiles within the cell body were captured at magnifications between 19,000X and 25,000X, ensuring all mitochondrial profiles were captured without duplication.

Image analysis was performed using ImageJ software version IJ1 following the method outlined by Schneider et al. (2012). (Schneider et al., 2012) Eight variables were analyzed per mitochondrial profile (Figure S3). The shape of each profile was categorized as round, elongated, or dumbbell‐in shape. Damage to cristae was assessed using a scale from 0 to 2 (0 indicating no damage, 1 slight damage, and 2 extensive damage; see Figure 3). The index score cristae derangement was calculated as (0 × N1 + 1 × N2 + 2 × N3) divided by (N1 + N2 + N3), where N represents the number of mitochondrial profiles in each of the three cells analyzed for a given plane. The area of each profile was measured in nanometers squared (nm^2^) after correcting for magnification using the free‐hand lasso tool in ImageJ. Proximity to the nucleus was measured in nanometers using a straight line from the outer mitochondrial membrane to the adjacent nuclear membrane. Mitochondrial membrane integrity was assessed by classifying each outer membrane as either intact or broken, with pinched membranes and swollen appearance also noted as binary observations (yes or no).

### 3.3 Single nuclei RNA sequencing

Single‐nuclei suspensions were prepared from dissected frozen LC tissue samples using the 10× Genomics Chromium Nuclei Isolation with RNAse inhibitor kit. Upon extraction of the LC tissue, it was immediately mixed with prechilled lysis buffer for tissue dissociation, with an optimized lysis time of 15 min. Subsequent steps for nuclei isolation followed the manufacturer's protocol.

To eliminate any nuclei clumps and aggregates, the nuclei suspension was passed through a 40 μm Scienceware® Flowmi™ Cell Strainer (Bel‐Art™ H136800040). Nuclei were then counted using a hemocytometer (C‐Chip 4 channel slides, NanoEntek) after staining with Trypan blue and ethidium bromide. Quality assessment was conducted using fluorescence microscopy (Invitrogen™ EVOS™ M5000 Imaging System). The nuclei concentration of samples was optimized to be within the range of 700–1200 nuclei/μl.

GEM Generation & Barcoding was carried out using the Chromium Next GEM Chip G and Chromium Controller. PCR cycle optimization ensured appropriate sample indexing and cDNA amplification, with 10 cycles utilized for cDNA amplification based on intended nuclei recovery. For cDNA library construction, 12–14 cycles were employed for sample index (Dual Index Plate TT Set A) PCR, taking into account the quantified cDNA input. Paired‐end libraries were sequenced on an Illumina Novaseq 6000 sequencer (Génome Québec, Canada).

The demultiplexed FASTQ files of 12 samples from two groups (AAV‐htauE14: 3 males +3 females and AAV‐GFP: 3 males +3 females) were aligned to the Rattus Norvegicus reference genome version mRatBN7.2 from Ensembl (Howe et al., 2021), using the 10x Genomics Cell Ranger version 7.2.0 pipeline, facilitating alignment, filtering, barcode, and UMI counting. The filtered count matrices of each sample were processed, examined, and visualized using Cellenics® community instance (https://scp.biomage.net/) hosted by Biomage Trailmaker, hosted by Parse Biosciences.

The emptyDrops method and cell size distribution filter were not applied as the data were already prefiltered. Nuclei with high mitochondrial content and a high probability of being a doublet (scDblFinder) (Germain et al., 2021) were filtered using different thresholds for each sample to ensure the retention of high‐quality nuclei for later analysis. Subsequently, the data were LogNormalized and combined into a single object for clustering analysis using the scTransform package in Seurat v4.4.0 (Hafemeister & Satija, 2019).

Clustering and dimensional reduction analysis (25 principal components) were performed using the Louvain clustering method (resolution = 0.8), and clusters were visualized using uniform manifold approximation and projection (UMAP) embedding with the Euclidean distance metric (minimum distance = 0.3). Clusters that did not contain nuclei from all replicates or exclusively from one replicate were excluded from downstream analyses.

A Wilcoxon rank‐sum test was conducted using Scanpy (Versions 1.10) to identify the top 25 differentially expressed genes (DEGs) within a cluster compared to all other clusters. Additionally, DEG analysis was conducted using the Presto package in R (Cephe et al., 2023), which employs the Wilcoxon rank‐sum test and area under the receiver operating characteristic (auROC) analysis to identify marker genes that distinguish different clusters. Finally, clusters were annotated based on these genes by comparing them with known cell type‐specific markers from previously published datasets.

Based on the expression of top marker genes, we identified various cell types, including GABAergic neurons (*Gad1* & *Gad2*: clusters 7, 21; *Gabrg3*: clusters 1, 5, 11, 16; *Gad1* & *Gabrg3*: cluster 8) (Yao et al., 2023; Zhou et al., 2023), glutamatergic neurons (*Slc17a7*: clusters 0, 2, 3) (Yao et al., 2023; Zhou et al., 2023), noradrenergic neurons (*Th* & *Dbh*, *Slc6a2*: cluster18) (Mulvey et al., 2018; Weber et al., 2024), oligodendrocytes (*Mog*: clusters 4, 6, 13) (Reiner et al., 2022), oligodendrocyte precursor cells (*Pdgfra*: cluster 12) (Ximerakis et al., 2019), microglia (*Arhgap*15 & *Ctss*: clusters 10, 20) (Ximerakis et al., 2019; Yao et al., 2023), astrocytes (*Slc1a2* & *Rorb*; clusters 9, 15) (Ximerakis et al., 2019; Yao et al., 2023), endothelial cells (*Ebf1*: clusters 17, 19) (Ximerakis et al., 2019; Yao et al., 2023), and ependymal cells (*Dynlrb2*: cluster: 22) (MacDonald et al., 2021) (Figure 3c). Cluster 14 was identified as neuronal, but its specific type could not be defined. Clusters 23 and 24 were unidentified due to the lack of nuclei from all replicates and the presence of multiple cell type markers, defining them as low‐quality clusters.

Differential expression analysis of the noradrenergic neurons between the two experimental groups was conducted using the pseudobulk limma‐voom package (Cephe et al., 2023), with genes considered differentially expressed if they had an adjusted p‐value (Adj‐*p*‐value) < 0.05 and an absolute log2 fold change >0.50. Gene ontology analysis was carried out using David Bioinformatics a previous report (Huang da et al., 2009).

### 3.4 Quantitative PCR (qPCR)

For qPCR analysis, LC extraction followed the same procedure as the WB method. Total RNA was extracted from the LC tissue using the Qiagen RNeasy isolation kit (RNeasy Micro Kit; Qiagen) following the manufacturer's protocol. RNA samples underwent DNAse I treatment (RNase‐Free DNAse Set 1500 Kunitz units; Qiagen) to eliminate genomic DNA. Primer sequences are indicated below.

**Table:** 

Gene	Primer sequence (forward)	Primer sequence (reverse)
*Ndufs1*	AGGAGATGCTGACCAACAATAG	GCATCACTCCACTGAATCCATA
*Ndufs2*	AGGAGATGCTGACCAACAATAG	GCATCACTCCACTGAATCCATA
*Sdha*	GAGTTGTAGGCAGGTGGATAA A	CTCACCCTCACACCAAGAATA C
*Mcu*	CCAGTTCACACTCAAGCCTAT C	CTTCTCCGCTTTCCTGCTAAT
*Slc8b1*	TAGACTGCACTGGCTCTTTG	CCCACACCAACCAGGATATT
*Atp5f1b*	GCTCAGAGGTATCTGCCTTATTG	CCTTCTTGGTGGTGGTGATT
*Mfn1*	GCAGCAGAGAAGAGGGTTTAT	CGTGACCTCCTTGATCTTCTTC
*Mfn2*	CAGGAGCAGCGGGTTTATT	CCTTTCCACTTCCTCTGTCATC
*Opa1*	ACTGACAAGCAGCTTCCTAATA	GTCATCGTGTTCCTTTCCTTTG
*Cacna1c*	CTACAACCAGGAGGGCATAATAG	CAT AGC GTC TTG CAT CCA GTA
*Cacna1d*	GAG TGG CCA TGG GTG TAT TT	GGC ATG CTA GTG TTT CGT TTG
*Dctn1*	GATGCTGAAGGTCTGGGTTT	ACTCAGCTCCTCTCCCTTAAT

The quality and concentration of total RNA were evaluated using the BioAnalyzer (Agilent 2100 BioAnalyzer) and the Nanodrop (Thermo Scientific; Nanodrop 1000 Spectrophotometer). RNA samples exhibiting clear bands for 18S and 28S, with 260/280 ratios between 1.8 and 2.1, and 260/230 ratios between 2.0 and 2.2, and RNA integrity number between 7 and 10 were considered suitable for further analysis. cDNA synthesis was performed using the Qiagen cDNA synthesis kit (QuantiTect Reverse Transcription Kit; Qiagen). For each qPCR reaction, a 10 μL PCR reaction volume was prepared, comprising 5 μL of SYBR Green Supermix (SsoAdvanced Universal; Bio‐Rad 1,725,270), 5 μM of each primer, 2.5 ng/nL of diluted cDNA, and 3.5 μL of RNAse‐free water, dispensed into a clear plastic 96‐well plate for three technical replicates. PCR cycling conditions included an initial denaturation step of 3 min at 95°C, followed by 40 cycles of 10 s at 95°C and 30 s at the optimal annealing temperature. A melting curve analysis was conducted to verify the specificity of primer amplification (Figure S5). Three technical replicates from each sample were included to control experimental errors.

### 3.5 Statistical analysis

All data are presented as mean ± standard error of the mean. Statistical analyses were conducted using OriginPro 2022b software. Hypothetical power analysis was based on a previous report (Omoluabi et al., 2021), where a power of 0.80 for Type II errors and 0.05 for Type I errors were assumed. ANOVAs of behavioral and molecular measures in this model suggest that 6–8 subjects per group (mixed sexes) will be sufficient to detect the predicted effects. For the EM aspects of this study, mitochondrial damage was assessed across three neurons per level with three randomly selected levels or depths across the extent of the LC in each animal. This generated mitochondrial profiles from nine neurons or an average total number of mitochondrial profiles of 561 per animal. Based on previous research (Weeks et al., 2007), this exceeds the number needed to produce a power of 0.80 with a medium effect size (*d* = 0.5). The sample size for qPCR was comparable with other studies measuring mitochondrial function (Márkus et al., 2016). Unpaired student t tests were used to compare two groups. One‐way ANOVAs with post hoc Tukey tests were used for comparisons involving more than two groups, with homogeneity of variance assumed (Levene's tests). Pearson correlation coefficient was used to measure the correlation. All data are included in the analysis.

## 4 RESULTS

### 4.1 Transduction of hyperphosphorylated human tau in the LC leads to deficits in spatial, and olfactory discrimination learning, impaired mitochondrial morphology and elevated LTCC Cav1.2 expression

We infused AAVs carrying CRE‐dependent htauE14, or htau or GFP into the bilateral LC of tyrosine hydroxylase (TH)‐CRE rats at 5 months of age and conducted a battery of behavioral tasks 10 months post‐infusion (Figure 1a). htauE14 was generated by mutating 14 S/T amino acids resides of the 0N4R human tau isoform to (Hoover et al., 2010) (Figure 1b), mimicking the abnormal phosphorylation observed in human AD brains (Hanger et al., 2009). Infusion of the AAVs resulted in 84.90 ± 2.72% (*n* = 5) transduction of the LC noradrenergic neurons (Figure 1c–e), and expression of human tau in these neurons (Figure 1f,g). htauE14 transduction also induced tau phosphorylation at S262 and S356 (Figure 1h,i), two microtubule binding sites involved in early tau pathology in AD (Ando et al., 2016). This suggests that htauE14 transduction activates tau kinases, leading to the phosphorylation of additional nonmutated sites. In a previous study, htauE14 infusion in the hippocampal CA1 region led to significantly higher levels of phosphorylated tau compared to htau, despite similar overall htau expression in the tissue (Crossley et al., 2024). This suggests that htauE14, unlike general human tau overexpression, is specifically associated with the activation of tau‐phosphorylating kinases.

While neither htau or htauE14 transduction induced changes in behavior in the open field (Figure 1 J1‐J3), elevated plus maze (Figure 1k), MBT (Figure 1l) and SPT (Figure 1m), htauE14, but not htau, caused impairment in the SLR test (*F*(2,22) = 17.839, *p* < 0.001) (Figure 1n). htauE14 rats exhibited significant lower discrimination index compared to htau and GFP rats (*p* < 0.01). Furthermore, htauE14 rats showed impaired odor discrimination of similar odors (*F*(2,22) = 4.088, *p* < 0.031) (Figure 1o).

**FIGURE 2:**
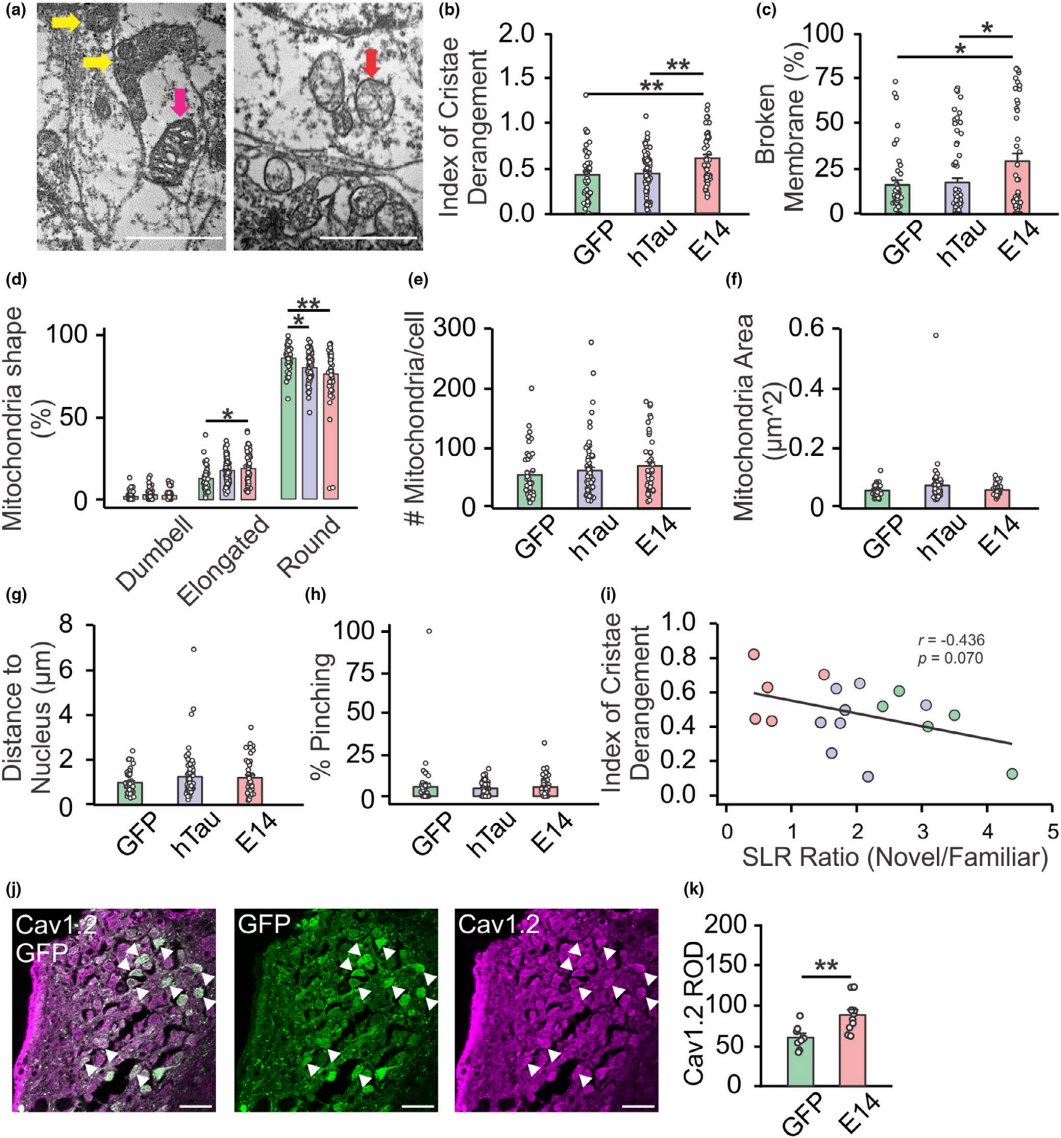
HtauE14 incubation in the LC leads to impaired mitochondrial morphology. (a). Example electron microscopy images of mitochondria showing intact (yellow arrows), mildly deranged (magenta arrow) and severely deranged (red arrow) mitochondria. Scale bars, 500 nm. (b) Index of cristae derangement. (c) Percentage of mitochondria with broken membranes. (d) Percentage of mitochondria of various shapes. (e) Numbers of mitochondria per cell. (f) Mean area of mitochondria per cell. (g) Distance to the nucleus. (h) Percentage of mitochondria showing pinching. (i) Correlation between cristae derangement and spontaneous location recognition (SLR) performance. (j) Examples images of L‐type calcium channel subunit Cav1.2 staining and GFP‐expressing cells. Scale bar, 50 μm. (k) Relative optical density (ROD) of Cav1.2 in GFP vs. htauE14 rats. *N* = 3F/2M (GFP), 7F/1M (htau) and 2F/3M (htauE14). *p < 0.05; **p < 0.01.

We next examined potential mitochondrial deficits associated with tau hyperphosphorylation using transmission electron microscopy (TEM). The analysis of TEM data was based on individual neurons in the LC. Each animal had nine neurons analyzed, with three cells per depth and three depths total. Figure S1 shows example mitochondria images. Comparing the levels of damaged mitochondrial cristae (Figure 2a illustrates intact and damaged cristae), a one‐way ANOVA revealed a significant main effect of the virus on cristae derangement levels (*F*(2,159) = 7.531, *p* < 0.001) (Figure 2b). htauE14 rats showed higher level of cristae derangement than other groups (*p* < 0.001). Furthermore, the htauE14 rats exhibited more mitochondria with broken membranes (*F*(2,159) = 4.902, *p* = 0.009) (Figure 2c). Mitochondria in htauE14‐transduced brains also displayed distinct morphology (*F*(4,477) = 10.241, *p* < 0.001) (Figure 2d). More mitochondria in the htauE14 animals exhibited an elongated shape compared to GFP rats (*p* < 0.05), whereas GFP animals had more round‐shaped mitochondria (*p* < 0.05 compared to htau; *p* < 0.01 compared to htauE14). No differences were observed among groups regarding the number of mitochondria per cell (Figure 2e), total mitochondrial area (Figure 2f), distance to the nucleus (Figure 2g), and percentage of pinching (Figure 2h). There was a nonsignificant trend indicating that cristae derangement was negatively correlated with spatial learning performance (*r* = −0.436, *p* = 0.070) (Figure 2i).

Concurrently, htauE14 transduction led to increased Cav1.2 expression in GFP^+^ neuron soma in the LC (*t*(16) = −3.125, *p* = 0.007) (Figure 2j,k). Heightened calcium influx through LTCCs has been associated with mitochondria oxidant stress in the LC (Sanchez‐Padilla et al., 2014).

### 4.2 htauE14 transduction leads to DEGs in a sex‐dependent manner

**FIGURE 3:**
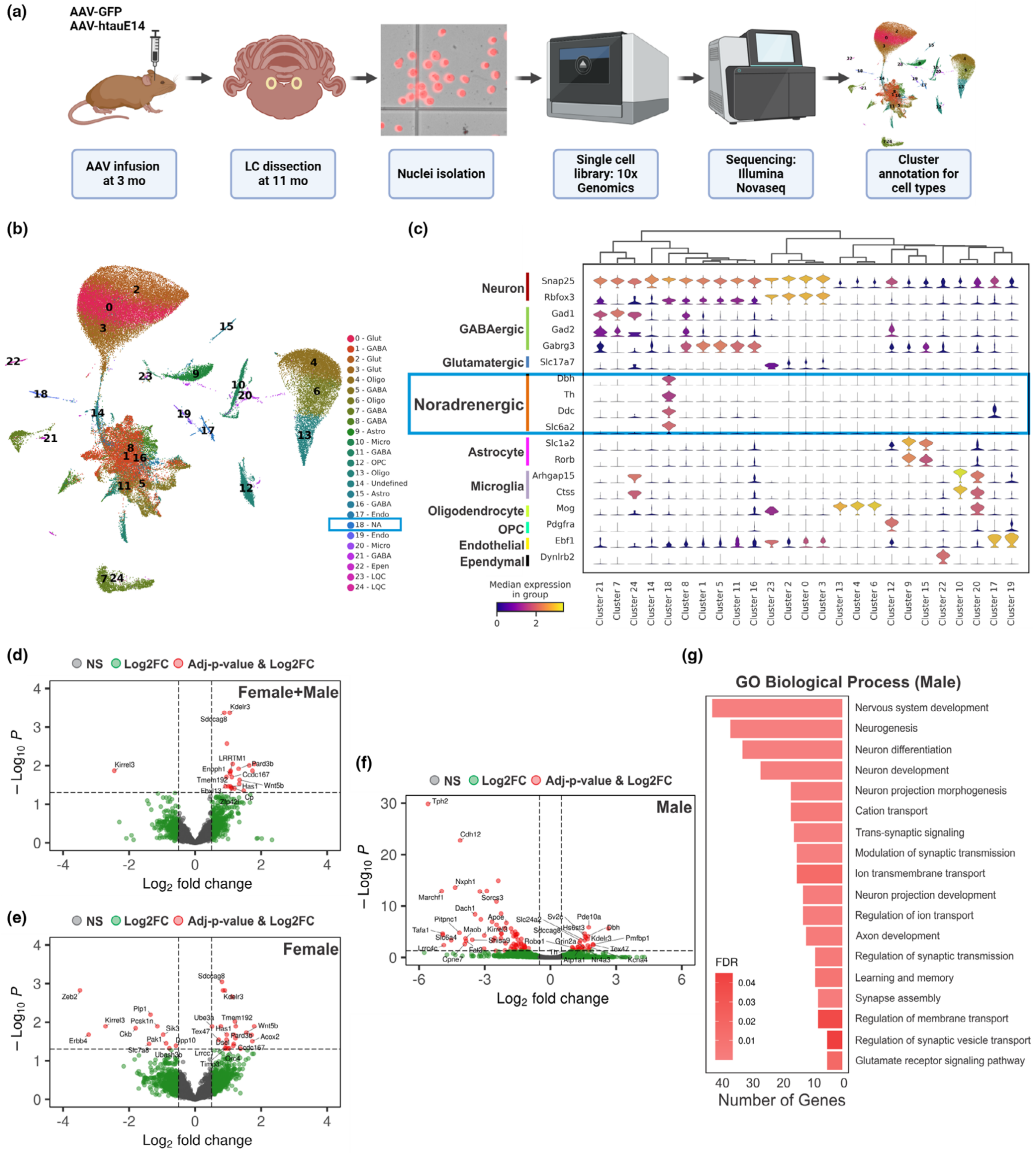
Single nuclei RNA sequencing demonstrates sex‐dependent differentially expressed genes in the LC induced by htauE14. (a) Schematic diagram demonstrating the experimental flow. (b) Cell‐type specific clustering of 76,178 nuclei from 12 samples (6 GFP and 6 htauE14, with 3 biological replicates for each sex). (c) Expression levels of canonical gene markers in each cell cluster. (d–f) Volcano plots displaying differentially expressed genes in all samples (d), female only (e) and male only (f) samples. Grey dots represent non‐significant (NS) data. Green dots indicate data with absolute log2 fold change (Log2FC) >0.50. Red dots represent data with adjusted *p*‐value (Adj‐*p*‐Val) <0.05 and absolute log2FC >0.50. F, female. M, Male. (g) Gene oncology (GO) analysis showing biological processes enriched in adult male htauE14 LC. FDR, false discovery rate.

In a separate cohort of 11‐month‐old rats that underwent AAV infusions at 3 months of age, we conducted single nuclei RNA sequencing (snRNA‐seq) to identify the DEGs of LC noradrenergic neurons induced by htauE14 at single‐cell resolution (work flow, see Figure 3a). Initially, after filtering out low‐quality nuclei, we obtained a high‐quality dataset comprising 76,178 nuclei from 12 samples (six GFP and six htauE14, with 3 three biological replicates for each sex). Clustering of the integrated dataset identified 25 clusters on a UMAP plot (Figure 3b), and was not influenced by batch effects (Figure S2). Using pan‐neuronal markers (*Snap25* and *Rbfox3*) (Dowsett et al., 2021; Gokce et al., 2016; Yao et al., 2023) and various canonical cell type markers, we identified neuronal and nonneuronal cell populations within the clusters (Figure 3c; Figure S3).

We focused on DEG analysis in the noradrenergic neurons (identified by *Th*, *Dbh*, *Ddc* and *Slc6a2*, cluster 18) to understand the direct effects of pretangle tau on these neurons. The DEG analysis of htauE14 vs. GFP revealed 18 upregulated and one downregulated gene (Adj‐p‐Val<0.05 and absolute log2FC >0.50) (Figure 3d). Upregulated genes are involved in diverse cellular functions such as synaptic organization (*LRRTM1* (de Arce et al., 2023)), cell polarity, differentiation and development (*Pard3b* (Thompson, 2022), *Sdccag8* (Tsutsumi et al., 2022), Wnt5b (Suthon et al., 2021)), metabolism (*Enoph1* (Zhang et al., 2016), *Has 1* (Maloney et al., 2022), *Cp* (Collins et al., 2010)), membrane transport and protein regulation (*Kdelr3* (Trychta et al., 2018), *Fbxl13* (Fung et al., 2018), *Tmem192* (Liu et al., 2012)), and transcriptional regulation (*Zfp42l* (Do Kim et al., 2011)). The downregulated gene, *Kirrel3*, is primarily involved in cell adhesion (Taylor et al., 2020). In the brain, decreased *Kirrel3* levels may disrupt proper neuronal adhesion and synaptic function (Taylor et al., 2020).

Subsequent analysis of htauE14 versus GFP in females (Figure 3e) and males (Figure 3f) revealed sex‐specific transcriptional differences: females had 22 upregulated and 11 downregulated genes, while males had 29 upregulated and 66 downregulated genes. Among these, only six genes show differential expression across both sexes: *Sdccag8*, *Kdelr3*, *and Tex47* (uncharacterized) were upregulated, while *Plp1* (myelin formation) (Kim et al., 2021), *Kirrel3* and *Erbb4* (cell proliferation and neural development) (Pitcher et al., 2022) were downregulated. Notably, in male htauE14 rats, besides neuron differentiation and development, a number of DEGs related to synaptic transmission, ion channel regulation and receptor signaling emerged (Figure 3g). Expression of several long noncoding RNAs were also affected. Table S1 summarizes the DEGs in overall and sex‐dependent comparisons.

### 4.3 Nimodipine chronic injection rescues behavioral deficits associated with htauE14

**FIGURE 4:**
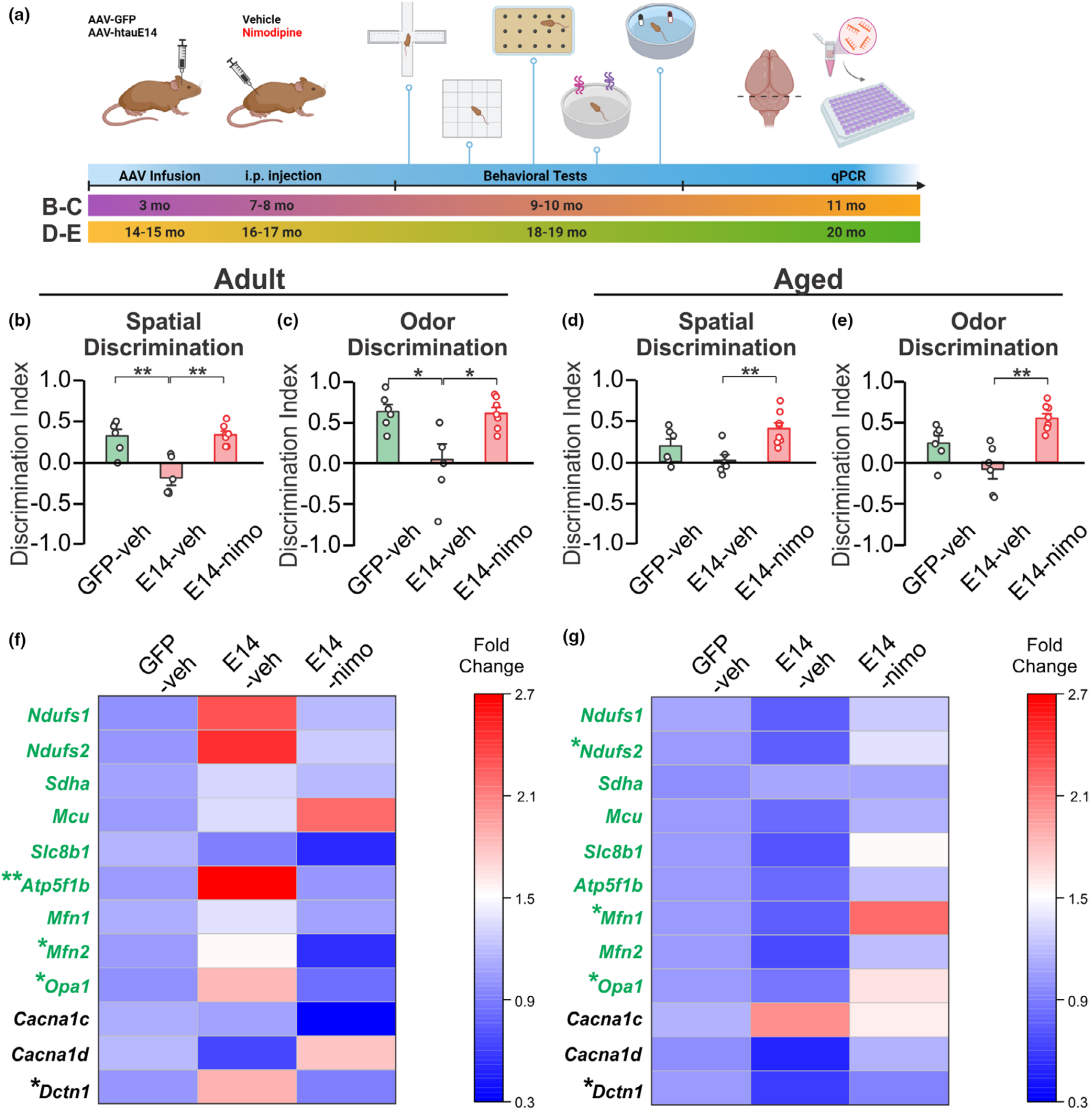
Chronic LTCC blockade by nimodipine prevents learning deficits and alters LC mRNA expression. (a) Schematic diagram demonstrating the experimental flow. (b, c) Spatial (b) and odor (c) discrimination index in adult rats. Veh, vehicle. Nimo, nimodipine. *N* = 3F/3M (GFP + Veh), 2F/4M (htauE14 + Veh) and 4F/4M (htauE14 + nimodipine). (d, e) Spatial (d) and odor (e) discrimination index in the aged rats. *N* = 4F/2M (GFP + Veh), 3F/3M (htauE14 + Veh) and 3F/4M (htauE14 + nimodipine). (f) Heatmap showing fold changes of various gene markers among groups in adult rats by qPCR measurement. Genes related to mitochondrial function are highlighted in green. (g) Heatmap showing fold changes of various gene markers among groups in aged rats by qPCR measurement. Genes related to mitochondrial function are highlighted in green. *N* = 3–6 for each group per marker, sex mixed. *p < 0.05; **p < 0.01.

We explored the potential therapeutic effects of LTCC antagonist nimodipine. LTCC blockers such as nimodipine have previously shown promise in improving cognitive function in dementia patients, likely due to their ability to cross the blood–brain barrier and modulate calcium influx into neurons (Ban et al., 1990; Disterhoft, Thompson, Moyer, & Mogul, 1996; Fischhof et al., 1989; Tollefson, 1990). Two age cohorts were employed here. In one cohort, rats were infused with AAVs at 3 months of age, followed by nimodipine injections at 7–8 months of age, for a duration of 8 weeks. The second cohort received AAV infusion at 14–15 months of age, followed by nimodipine injection 2 months later for the same duration (Figure 4a). In the cohort infused at 3 months, htauE14 rats injected with vehicle exhibited spatial learning deficits, which were prevented by nimodipine injection (*F*(2,16) = 12.425, *p* < 0.001) (Figure 4b). Similar results were observed in the odor discrimination test (*F*(2,16) = 7.176, *p* = 0.006), where nimodipine injection prevented the odor discrimination deficit associated with htauE14 animals (Figure 4c). In the aged cohort, significant effects of AAVs were observed on spatial discrimination learning (*F*(2,17) = 6.921, *p* = 0.006) (Figure 4d) and olfactory discrimination (*F*(2,17) = 13.424, *p* < 0.001) (Figure 4e). The nimodipine group demonstrated better performance than htauE14 rats with vehicle infusion. General behavior was not affected by nimodipine injections (Figure S4).

We measured mRNAs related to mitochondrial function (Cuperfain et al., 2018), including *Mcu* (mitochondrial calcium uniporter), *Ndufs1* and *Ndufs2* (Complex I subunits), *Sdha* (Complex II subunit), *Atp5f1b* (mitochondria ATP synthase), *Mfn1*, *Mfn2* and *Opa1* (encoding mitochondrial fusion proteins), as well as *Cacna1c* and *Cacna1d* (LTCC subunits) (Crossley et al., 2023) and Dctn1 (dynactin) (Konno et al., 2017). Strikingly, distinct mRNA patterns emerged between adult and aged rats (Figure 4f,g; Table S2). In adult rats, *Mfn2* (*F*(2,11) = 4.587, *p* = 0.036), *Opa1* (*F*(2,12) = 5.262, *p* = 0.023), *Atp5f1b* (*F*(2,11) = 8.219, *p* = 0.007) and *Dctn1* (*F*(2,12) = 6.458, *p* = 0.012) were significantly different among groups (Figure 4f). The expression levels of these markers were elevated by htauE14 and were reversed by nimodipine injection (*p* < 0.05). In aged rats, *Ndufs2* (*F*(2,12) = 4.781, *p* = 0.03), *Mfn1* (*F*(2,11) = 4.359, *p* = 0.040), *Opa1* (*F*(2,11) = 4.273, *p* = 0.042) and *Dctn1* (*F*(2,10) = 4.326, *p* = 0.044) showed significant differences among groups (Figure 4g). Nimodipine injection elevated the expression levels of *Ndufs2*, *Mfn1*, and *Opa1* compared to vehicle‐injected htauE14 rats (*p* < 0.05), whereas htauE14 vehicle rats had significantly lower expression of *Dctn1* (*p* < 0.05), which was reversed by nimodipine injection.

There were no significant changes in the mRNA levels of the LTCC subunits *Cacna1c* and *Cacna1d* with htauE14 transduction. In aged rodents, the calcium dysregulation in the hippocampal CA1 occurs due to increased membrane expression without changes in LTCC mRNA levels (Nunez‐Santana et al., 2014). Our results suggest that pretangle tau‐induced LTCC alterations also occur at the protein level. In another report (Guzman et al., 2018), chronic LTCC blockade with isradipine diminished mitochondrial oxidant stress in substantia nigra dopamine neurons, without altering LTCC mRNA expression, consistent with our results.

## 5 DISCUSSION

Pretangle tau pathology in the LC represents one of the earliest opportunities for potential intervention in AD. In this study, we explored the mechanisms behind pretangle tau toxicity in the LC, which result in axonal degeneration and the learning deficits observed in both our current research and previous studies (Ghosh et al., 2019; Omoluabi et al., 2021). Single nuclei transcriptomics revealed changes in multiple genes linked to cell proliferation and differentiation, metabolic signaling, stress response, synaptic function and ion channels, showing a pattern dependent on sex. Furthermore, tau hyperphosphorylation resulted in distorted mitochondrial cristae and elevated expression of the LTCC Cav1.2 in LC soma. These pathological shifts coincided with impairments in spatial and olfactory discrimination learning. Interestingly, chronic blockade of LTCCs for 8 weeks mitigated behavioral deficits linked to hyperphosphorylated tau and modified mitochondrial mRNA expression, suggesting a possible role of LTCC‐mediated mitochondrial dysfunction in pretangle tau pathology.

LC neuronal health plays a crucial role in aging and AD. Postmortem studies have linked higher numbers of LC neurons to better cognitive performance and slower cognitive decline before death (Kelly et al., 2017; Theofilas et al., 2017; Wilson et al., 2013). Recent MRI evidence has revealed that the deterioration of LC integrity precedes the accumulation of tau in the medial temporal lobe and correlates with diminished cognitive performance, highlighting the significance of LC integrity as an early biomarker for preclinical AD (Bueichekú et al., 2024). In AD brains, significant neuronal loss in the LC correlates with changes in mRNAs regulating mitochondrial function (Kelly et al., 2017). Moreover, augmentation of LC norepinephrine activity has been proposed as an intervention in AD. Recent research has demonstrated that novelty‐related LC activity is associated with lessened tau deposition in the entorhinal cortex and slower memory decline in humans (Prokopiou et al., 2023). Findings from the LC pretangle tau model are consistent with these human studies. LC axonal degeneration in the hippocampus and olfactory cortex are concomitant with spatial and olfactory learning deficits (Ghosh et al., 2019; Omoluabi et al., 2021), which are reversed by novelty‐associated LC phasic stimulation (Omoluabi et al., 2021).

Our findings suggest that pretangle tau‐induced LC axonal degeneration may result from mitochondrial dysfunction mediated by LTCCs. Previous research has reported LTCC‐mediated mitochondrial oxidant stress in the LC (Sanchez‐Padilla et al., 2014). Stressors such as hypoxia can activate LTCCs on the cell membrane, leading to calcium influx, which then moves into the endoplasmic reticulum (ER). Through mitochondrial‐associated membranes (MAMs), calcium released from the ER enters mitochondria via the mitochondrial calcium uniporter, causing oxidant stress. The resultant oxidant stress is prevented by an LTCC blocker, isradipine (Sanchez‐Padilla et al., 2014). Dysregulation of mitochondrial calcium homeostasis is implicated in AD pathology (Walker & Moraes, 2022), impacting various cellular functions. Increased calcium influx into neurons via LTCCs (Coon et al., 1999; Disterhoft et al., 1994), and altered MAM contact or mitochondrial ion exchanger expression can elevate mitochondrial calcium levels (Jadiya et al., 2019). This elevation stimulates ROS production, reduces ATP synthesis, and activates apoptotic pathways (Jadiya et al., 2019; Rizzuto et al., 2012).

Some evidence suggests that hyperphosphorylated tau is associated with increased LTCC currents or hyperfunction in hippocampal CA1 neurons (Crossley et al., 2024; Furukawa et al., 2003; Wang & Mattson, 2014). Here we demonstrated that learning deficits induced by LC hyperphosphorylated tau were accompanied by an upregulation of LTCC expression in LC noradernergic neurons and mitochondrial deficits. The decreased expression of the *Slc8a1* gene, encoding the Na^+^/Ca^2+^ exchanger 1 (NCX1) at the plasma membrane (Khananshvili, 2013), was observed in male adult htauE14 rats. This could also exacerbate elevated intracellular calcium levels. Hyperphosphorylated tau may enhance LTCC function via Src family kinases, such as Src or Fyn (Crossley et al., 2023). Additionally, it can elevate mitochondrial calcium levels by inhibiting calcium efflux through its effect on the mitochondrial Na^+^/Ca^2+^ lithium exchanger (NCLX) (Britti et al., 2020). However, *Slc8b1*, the mRNA encoding NCLX, was not found to be significantly different in the htauE14 groups in our study.

Conversely, mitochondrial signals, including calcium, ROS, and ATP, can initiate nuclear transcriptional changes, influencing various cellular functions including cell proliferation, apoptosis, mitogenesis, metabolism and immunomodulation (Celsi et al., 2009; Rizzuto et al., 2012; Walker & Moraes, 2022). In the LC of female adult rats, several genes associated with these functions were upregulated. For instance, *Has1*, a hyaluronan synthases (Lee & Spicer, 2000), and *Enoph1*, an enzyme involved in methionine synthesis (Wang et al., 2021), are widely implicated in cell proliferation and stress responses. *Timp3*, encoding a tissue inhibitor of metalloproteinases 3, plays a role in regulating extracellular matrix remodeling and cell proliferation (Cabral‐Pacheco et al., 2020). *Ube3a*, encoding an ubiquitin‐protein ligase, participates in protein degradation pathways, including those involved in apoptosis regulation (Lopez et al., 2018). *Acox2*, or peroxisomal acyl‐CoA oxidase 2, contributes to fatty acid metabolism (Neupane et al., 2022), which is essential for mitogenesis, metabolic function and providing substrate for mitochondrial energy production (Eling & Glasgow, 1994; Neupane et al., 2022). *Gyg1*, encoding an enzyme catalyzes glycogen synthesis (Laforet et al., 2017), was also elevated here by htauE14. Additionally, *Kdelr3*, encoding an ER protein retention receptor protein and up‐regulated in both sexes, plays a critical role in protein trafficking and can regulate ER stress (Trychta et al., 2018), thereby indirectly influencing mitochondrial function. Conversely, genes such as *Robo1* (Koohini et al., 2019) and *Hs6st3* (Sikora et al., 2016), which are implicated in immune response and inflammation, were found to be down‐regulated in male adult htauE14 rats. Interestingly, *Dbh* and *Th*, which encode enzymes responsible for norepinephrine production (Nagatsu, 1991), were upregulated in male rats, suggesting a compensatory effect for LC axonal degeneration observed in this model (Ghosh et al., 2019; Omoluabi et al., 2021). Alterations of these genes likely mediate adaptive cellular responses to stress in the pretangle tau brains.

The proper functioning of respiratory chain complexes relies on intact mitochondrial ultrastructure and the integrity of the inner mitochondrial membrane (Cogliati et al., 2016). Cristae derangement observed in htauE14 brains likely results in reduced biogenesis. *Ndufs2*, which encodes a subunit of mitochondrial respiratory Complex I, was reduced by htauE14 transduction in aged (20‐month‐old) rats but showed a trend of elevation in adult rats (11‐month‐old). Dysregulated mitochondrial dynamics such as altered fusion and fission rates disrupt cellular function and viability (Schulz et al., 2012). In htauE14 brains, mitochondria appeared more elongated, indicating excessive fusion in the LC, consistent with findings in other mutant tau models (Eckert et al., 2014). Fusion promotes the sharing of metabolites and mitochondrial DNA, serving as a complementary regulation when mitochondria are damaged (Lin et al., 2022). Interestingly, mRNAs involved in mitochondria fusion (*Mfn1, Mfn2* and *Opa1*) (Lin et al., 2022) were higher in the adult rats but lower in the aged htauE14 brains. Nimodipine injection appeared to have normalized their expression. *Marchf1*, encoding an E3 ubiquitin‐protein ligase (Ishido et al., 2009), was found to be down‐regulated in male adult htauE14 rats. This protein could impact mitochondrial dynamics and mitophagy in response to ER stress (S. Lee et al., 2022). Distressed or damaged mitochondria may in turn trigger enhanced mitophagy and reduced mitochondria transport along cellular processes.

Additionally, phosphorylated tau has been shown to disrupt anterograde transport of specific cargos, including mitochondria (Ittner et al., 2009). *Dctn1*, encoding a major P150 subunit of dynactin protein complex crucial for axonal transport (Konno et al., 2017), was increased in adult htauE14 rats and reversed by nimodipine injection. An opposite pattern emerged in aged rats. The opposite patterns of mitochondrial and axonal transport markers in adult and aged rats suggest strong compensatory effects in younger rats to pretangle tau stress, which are not the case in aged rats. Nevertheless, nimodipine injection reversed many of the changes in both age groups. Together, abnormally phosphorylated tau can contribute to impaired mitochondrial function and neuronal degeneration through both calcium‐dependent and independent pathways.

Sex differences, often overlooked in preclinical AD studies, are evident in AD across various parameters, including mitochondrial function (Grimm & Eckert, 2017; Laws et al., 2018; Mosconi, Berti, Guyara‐Quinn, et al., 2017; Mosconi, Berti, Quinn, et al., 2017; Podcasy & Epperson, 2016; Yanguas‐Casas et al., 2018). As individuals age, mitochondria show significant sexual dimorphism, particularly in oxidative capacities, calcium handling, and resilience to oxidative stress. Males are more vulnerable to age‐related mitochondrial dysregulation (Borrás et al., 2010), while estradiol in females provides protection (Borrás et al., 2010). However, vulnerability in females emerges during perimenopause (Mosconi, Berti, Quinn, et al., 2017), and women exhibit more pronounced tau pathology than men (Tsiknia et al., 2022). In animal models, female P301L tau mutant mice display heightened pathological changes, likely associated with synaptic deficiencies (Buccarello et al., 2017). One limitation of our study is the moderate sample size, limiting the power of sex‐specific statistical analyses. Our transcriptomic analysis, revealing substantial sex dimorphism in DEGs in htauE14 brains, supports the notion that AD, including tau pathology, exhibits sex dimorphism. Particularly, in male htauE14 rats, a number of genes related to synaptic function and ion channels were downregulated. How this relates to sex‐dependent learning deficits requires future investigation.

In summary, this study uncovers the mechanisms behind pretangle tau toxicity in the LC, leading to axonal degeneration and cognitive deficits. Tau hyperphosphorylation correlates with mitochondrial dysfunction and LTCC overexpression. Targeting LTCC‐mediated calcium overload and mitochondrial dysfunction holds therapeutic promise in pretangle tau pathology, as evidenced by our nimodipine experiment.

## AUTHOR CONTRIBUTIONS

QY conceived the project idea. TM, ZH, JEP, ARSF, ACWW conducted experiments. TM, ZH, JEP, ACWW, TB, QY analyzed the data. JJED, SGW, TB, QY provided funding or resources. TM, ZH, JEP, QY wrote the first draft. TM, ZH, JEP, JJED, SGW, ACWW, TB, QY edited the manuscript.

## FUNDING INFORMATION

This work was supported by a Canadian Institutes of Health Research project grant to QY and TB (#PJT‐169197), and an Alzheimer Society of Canada New Investigator Grant to SGW (#20–04).

## CONFLICT OF INTEREST STATEMENT

The authors report no competing interests.

## Supporting information


Data S1.

## Data Availability

All data are included in the manuscript. The snRNAseq dataset is available from the GEO database under the accession number GSE272794.
