# Supplementary material for: Locus coeruleus vulnerability to tau hyperphosphorylation in a rat model
Source: Aging Cell. 2024 Nov 9;24(3):e14405. doi: 10.1111/acel.14405 (PMC11896524; doi:10.1111/acel.14405)
Supplement: Supplementary file 1 — Data S1. [file ACEL-24-e14405-s001.pdf]

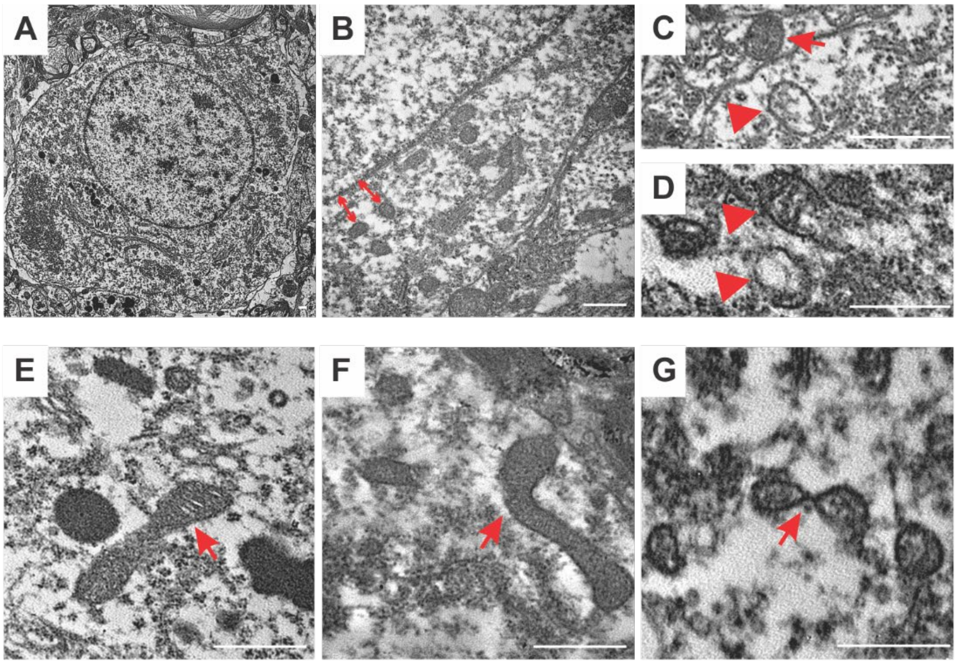

Supplementary Figure 1. Example images of mitochondria under electron microscopy.
A. Whole cell with nucleus. **B.** Proximity to the nucleus measurement (arrows). **C.** A healthy round mitochondria (arrow) and one with cristae derangement (arrowhead). **D.** Mitochondria with broken membrane (arrowheads). **E.** Dumbbell-shaped mitochondria (arrow). **F.** Elongated mitochondria (arrow). **G.** Mitochondria pinching (arrow). Scale bars, 500 nm.

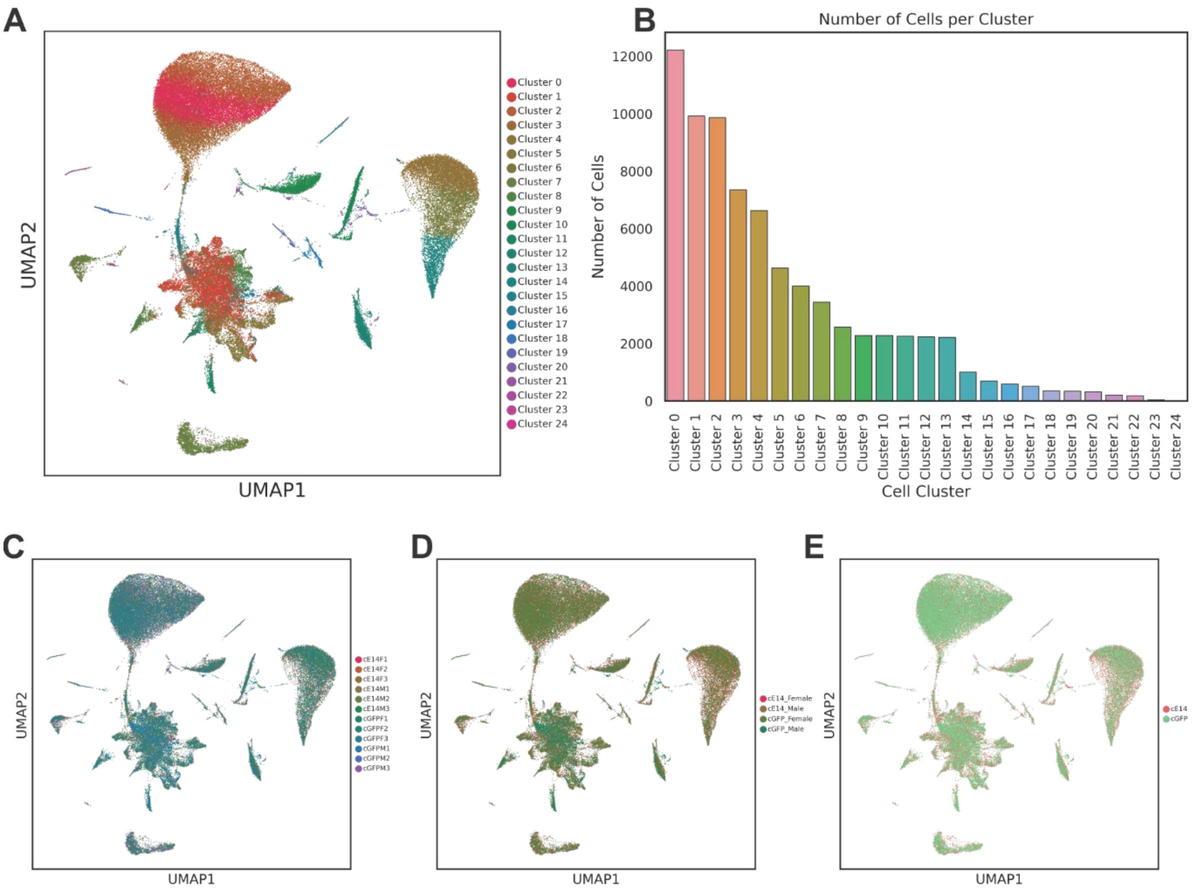

Supplementary Figure 2. Locus coeruleus cell clustering was not influenced by batch effects. **A.** UMAP project of the 25 clusters of 76,187 nuclei from 12 samples. **B.** Bar plot showing the number of cells per cluster. **C-E.** UMAPs demonstrate the integration of data at individual sample (C), sex (D) and group (E) levels.

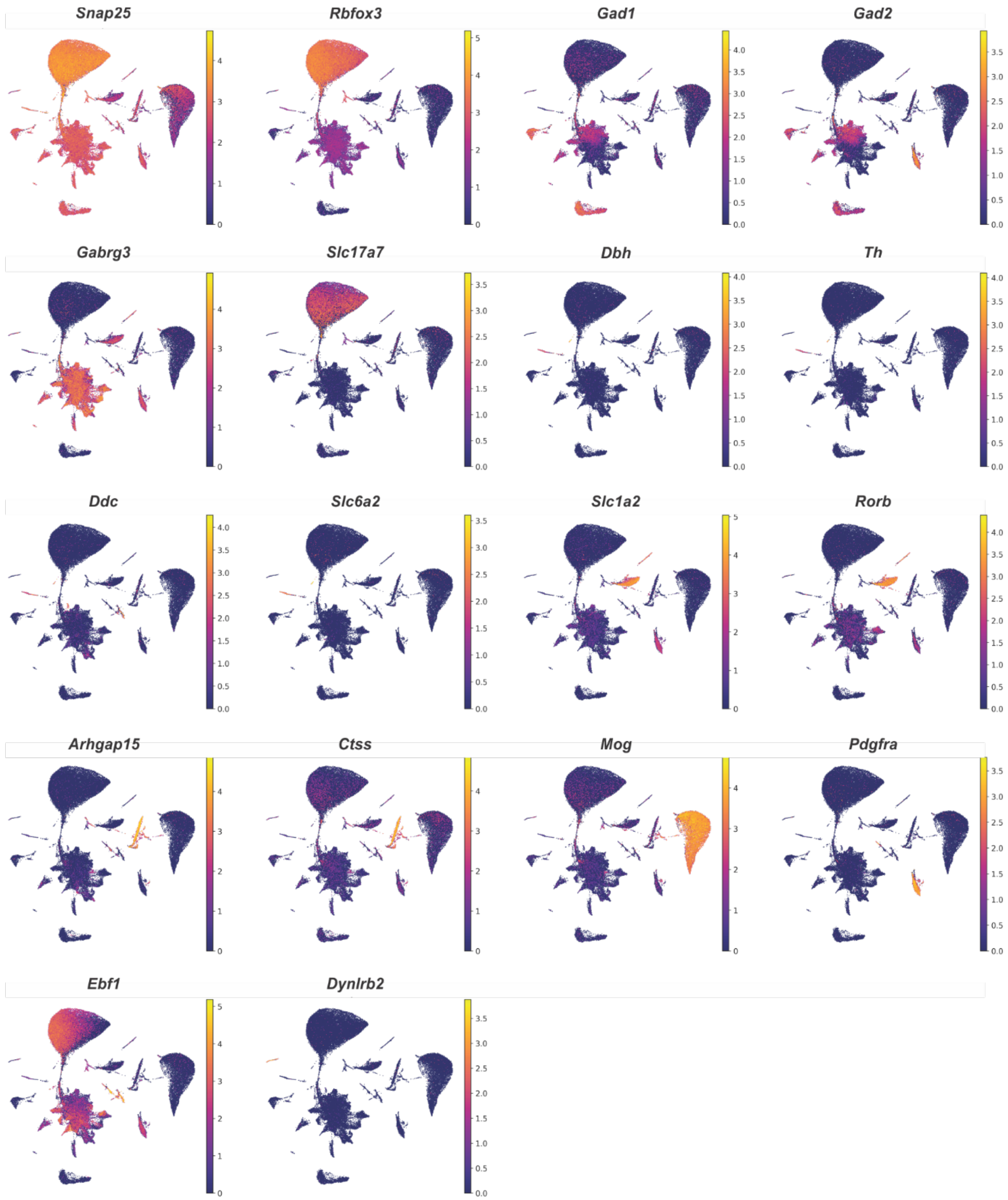

Supplementary Figure 3. Feature plots highlight the expression of representative common cell type-specific gene markers.

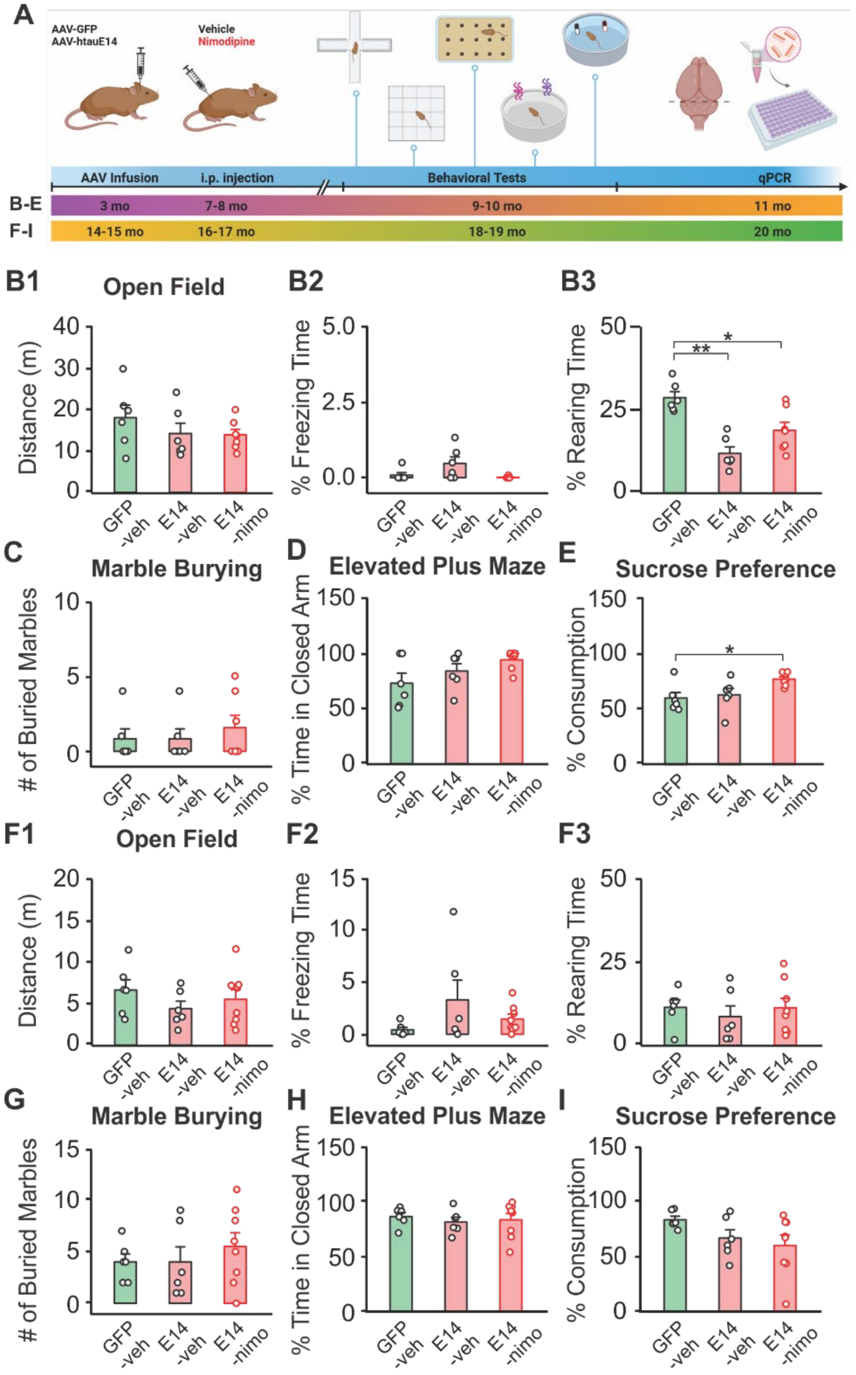

Supplementary Figure 4. The effects of chronic nimodipine injection on general behavior. **A.** Schematic diagram illustrating the experimental flow. **B1-B3.** Open field tests for the 3 month-injected cohort, including distance traveled (B1), percentage of time spent freezing (B2) and percentage of time spent rearing (B3). **C.** Number of marbles buried by the 3 month-injected cohort. **D.** Percentage of time spent in the closed arm of an elevated plus maze by the 3 month-injected cohort. **E.** Sucrose water consumed by the 3-month cohort. **F1-F3.** Open field tests for the 14-15 months-injected cohort. **G.** Number of marbles buried by the 14-15 months-injected cohort. **H.** Percentage of time spent in the closed arm of an elevated plus maze by the 14-15 month-injected cohort. **E.** Sucrose water consumed by the 14-15 month-injected cohort. Veh, vehicle. Nimo, nimodipine. N = 3F/3M (GFP+Veh), 2F/4M (htauE14+Veh) and 4F/4M (htauE14+nimodipine). $*p < 0.05$, $**p < 0.01$.

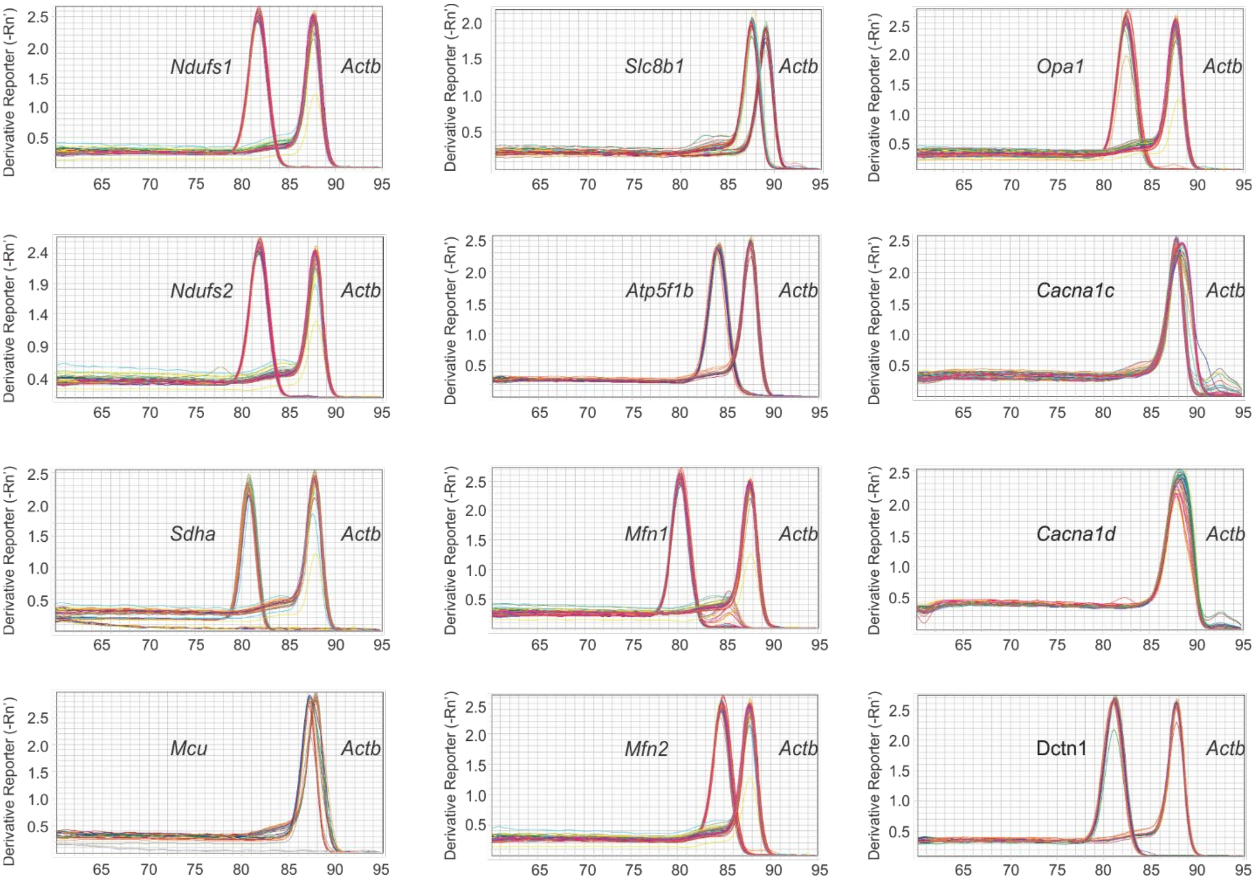

Supplementary Figure 5. Melt curves for qPCR primers.

E14 vs cGFP			E14 vs GFP (Female)			E14 vs GFP (Male)			
Upregulated Genes (22)		Downregulated Genes (1)	Upregulated Genes (25)		Downregulated Genes (11)	Upregulated Genes (30)		Downregulated Genes (68)	
coding gene (18)	LncRNA (4)	coding gene	coding gene (22)	LncRNA (3)	coding gene	coding gene (29)	LncRNA (1)	coding gene (66)	LncRNA (2)
Kdelr3, Sdccag8, Pard3b, LRRTM1, Enoph1, Ccdc167, Wnt5b, Has1, Fbxl13, Tmem192, Cp, Zfp42l, ,ENSRNOG00000038987, ENSRNOG00000063779, ENSRNOG00000033449, ENSRNOG00000065049, ENSRNOG00000000167, ENSRNOG00000064129	ENSRNOG00000069879, ENSRNOG00000070128 (LOC120095539), ENSRNOG00000064128, ENSRNOG00000070433	Kirrel3	Sdccag8, Kdelr3, ENSRNOG00000037911, ENSRNOG00000065049, ENSRNOG00000038987, Tmem192, Ube3a, Wnt5b, Has1, ENSRNOG00000015637 (LOC690507, Bpifb5, Bpifb9), Pard3b, ENSRNOG00000063779, Tex47, Acox2, Ccdc167, Dsel, ENSRNOG00000067040 (Zfp442), Timp3, Orc4, ENSRNOG00000032195, Lrrcc1, ENSRNOG00000064129	ENSRNOG00000062963, ENSRNOG00000070128 (LOC120095539), ENSRNOG00000069879,	Zeb2, Plp1, Pcsk1n, Kirrel3, Ckb, Erbb4, Sik3, Pak1, Slc7a8, Dpp10, Ubash3b	Pde10a, AABR07029272.1 (Dock2), Hs6st3, Sv2c, Dbh, Kdelr3, ENSRNOG00000038987, Slc24a2, Sdccag8, AABR07044900.1 (Ctnna3), Pmfbp1, Tex47, AABR07049554.2 (coding gene), Kcna4, Etl4, Hdac9, Dpp10, Robo1, Fgf13, Tmem163, Grin2a, ENSRNOG00000062930 (coding gene), Atp1a1, Pdlim5, LOC103692025(ENSRNOG00000066703, coding gene), Nr4a3, AC125757.1, Oprm1, Th	ENSRNOG00000070939	Tph2, Cdh12, Opcml, Nxph1, Csmd3, Marchf1, Sorcs3, Nlgn1, Sgcz, Dach1, Brinp3, Hs6st2, Apoe, Olfm3, Slc8a1, Schip1, Cntn3, Ncam2, Edil3, Pitpnc1, Tafa1, Hpse2, Fam13a, Epha6, Nyap2, Lcorl, Kirrel3, Stard13, Mbp, Tle4, Il1rapl2, Maob, Egfem1, Car10, Shisa9, Slc6a4, Vwc2, Fat3, Ntm, Fgf1, Dnm3, Zfhx3, Actb, Cpne7, S100b, Lrrc4c, Cntn1, Stxbp5l, Unc5c, Abat, Sorbs2, Dscam, Cachd1, Rora, Gria4, Ndrg2, Myo16, Aff3, Erbb4, Kcnd2, Cdyl2, Glul, Kif26b, Plp1, Ldhb, Ndufb7	ENSRNOG00000070379, ENSRNOG00000068121

Supplementary Table 1. Differentially expressed genes

	Adult		Aged	
**Gene**	**Degree of freedom**	***p* value**	**Degree of freedom**	***p* value**
Ndufs1	$F(2,12) = 2.631$	0.113	$F(2,12) = 2.192$	0.154
Ndufs2	$F(2,12) = 2.690$	0.108	$F(2,12) = 4.781$	0.03*
Sdha	$F(2,7) = 0.185$	0.835	$F(2,12) = 0.079$	0.925
Mcu	$F(2,10) = 2.060$	0.178	$F(2,11) = 1.287$	0.315
Nclx	$F(2,13) = 1.279$	0.311	$F(2,10) = 1.320$	0.31
Atp5f1b	$F(2,11) = 8.219$	0.007**	$F(2,11) = 1.268$	0.32
Mfn1	$F(2,11) = 0.127$	0.882	$F(2,11) = 4.359$	0.04*
Mfn2	$F(2,11) = 4.587$	0.036*	$F(2,12) = 2.122$	0.163
Opa1	$F(2,12) = 5.262$	0.023*	$F(2,11) = 4.273$	0.042*
Cacna1c	$F(2,10) = 2.656$	0.119	$F(2,10) = 0.373$	0.698
Cacna1d	$F(2,11) = 2.756$	0.107	$F(2,8) = 3.432$	0.084
Dctn1	$F(2,12) = 6.458$	0.012*	$F(2,10) = 4.326$	0.044*

Supplementary Table 2. qPCR marker gene statistics
